# A Few Weeks Abroad—Notes by the Way

**Published:** 1853-01

**Authors:** W. H. Dwinelle


### CORRESPONDENCE.

### *A Few Weeks Abroad—Notes by the Way.*

The city of Paris! A few years ago, the highest degree of skill and the greatest advancement in our profession, was almost by general acknowledgment conceded to Paris. In our own country in those times, the French dentist was the dentist *par excellence.* There was a magic in his foreign name more potent than the recommendations of all the country parsons, and ex-justices, that ever graced an aspiring dentist's first card. Now, the most popular, the most skillful and most successful dentist in Paris is an American. Years ago, Dr. Brewster, in his own person, established this reputation for America, and maintained it, until retiring with a large fortune, Dr. T. W. Evans succeeded to his position, bringing with him high proficiency in our art, together with the latest American improvements.

We speak with all deference to the worthy names of Jourdain, Fauchard, Duval, Baumes, Delabarre, Desirabode and others, yet still persistingly point to practical results.

We find Dr. Evans at the place formerly occupied by Dr. Brewster, 15 *Rue d' la Paix;* a very desirable location, central yet retired, about midway from the Boulevards on the one side, and the Palace of the Tuilleries on the other.

The operating and reception rooms of his office are most conveniently arranged, the latter are several in number, and are exceedingly useful in separating *classes* of patients, a consideration of indispensable importance growing out of the customs of the country; besides, too, the delicacy and shyness of, and the studious concealment with which our patients formerly cloaked the necessity of a visit to a dentist, exists here, often to the most extreme degree. Many a fair one with a complete set of artificial dentals, would, on interrogation, be totally *oblivious* to even the existence of a profession like ours! Dr. Evans told me that on some occasions he had had the husband and wife in different reception rooms at the same time, awaiting his services, while he was under the strongest injunctions of secrecy from each not for the world to let "my dear" know their teeth were defective, or that they had been at his office.

Dr. E's method of doing business exhibits a fine illustration of the advantages of system and order, elaborated to no common degree of perfection, without it, it would be impossible for him to accomplish the vast amount of labor which daily passes through his hands. His hours are divided off and set apart for specific purposes, as distinctly as the divisions on the face of a dial—the morning, the mid-day and the evening hours; hours for consultation and making appointments, hours for operative, and hours for mechanical dentistry.

The art of making the most of one's time is a great art, the ability to use all of the fragments and intervals, as well as the hours of time to the greatest possible advantage, is worthy the study and may tax the powers of the highest order of intelligence. The English excel us in order, we them in activity. It is only when the two characteristics are combined that the perfection of dispatch is realized. Slow plodding order is continually accomplishing something, mere activity often only frets and chafes against itself. "There is a wide difference between quickness and dispatch."

Dr. Evans is quite a favorite with Louis Napoleon, which of course brings him a large share of court patronage. In addition to this he operates for most of the crowned heads of Europe, besides other distinguished individuals in great numbers. A few days previous to my call upon the doctor, Jenny Lind and her husband had been inmates of his house for several days, she being his patient during the time.

His method of operating as I have before intimated, is American, though not always with that deliberation and finish which would be so gratifying to himself. This he pleads is impossible for him to do, owing to the multiplicity of his engagements. In this connection, I must not forget to state, that the unknown exhibitor mentioned in an article in the July number of the Journal, on the dental exhibition of the World's Fair, "—— *Philadelphia, No.* 558," whose pluggings are stated to be the most beautiful the writer ever saw, was Dr. Evans of Paris. Though the doctor has entirely banished the use of the Crawcour, as well as his own cadmium amalgam from his practice, he still persists in the hope that some good may yet "come out of Nazareth," and with a French chemist for his aid, is again experimenting with cement; he showed me a stopping of his new cement, which had been in a tooth in his own mouth for more than a year. Its appearance was certainly very satisfactory, the borders of the stopping were clean, distinct and solid, and the tooth bone or enamel was not discolored in the least. Gathering a lesson of prudence from the past, the doctor refuses either to publish or to use it generally until time has tested it more fully.

In Paris are many "cunning workers in metals"—men who have served years of apprenticeship in the manufacture of jewels and the most delicate mechanisms, men greatly accomplished by skill and experience, and yet they can be hired at comparatively low wages. Several of these Dr. Evans has secured in his laboratory to his great advantage. Some of these workmen will take a thick piece of gold plate, and by hammering, stretching, driving and stamping, will make it almost accurately fit a metallic model without swaging; a few blows upon the swages with the plate between, secures entire accuracy. Even though there should be three or four clasps to the plate, they are all worked up out of the plate, entirely doing away with the necessity of soldering, except in attaching the teeth to the plate. His mechanical work compares favorably with the best executed here. I was sorry, however, to see in Paris, what I also noticed in London, a willingness on the part of our profession to bend to the dictation of patients, especially with reference to the mechanical department, and the prior operations connected with it. The practice of covering diseased roots and ulcerating gums with costly dental fixtures is quit too common. The plea that the patient *will not* have the mouth properly prepared for the reception of artificial teeth, is hardly a sufficient one. I have no means of knowing why the operation of extracting the teeth should be more painful to a Frenchman or an Englishman than to an American; and yet our patients do universally submit to it rather than wear a dental appliance which would be discreditable alike to dentist and the patient. Men of world-wide reputation in our profession, especially, ought to have the control of their method of practice in their own hands. What would Velpeau say to the patient who would dictate to him how to operate for the cataract of his eye; and how would Liston brook the impudence of the subject, who would undertake to instruct him how to amputate his limb. The moment my patient assumes the dentist, that moment I yield him the whole ground, and consider that my consultations and my services are alike useless to him; not but there are points upon which it is proper and necessary for me to confer with my patient, but when he invades the sanctuary of my principles of practice, established by years of experience, and the combined verdict of my profession, and desires me to go counter to them, then my course of duty is plain, I can better afford to lose my patient, than lose the self-respect I should part with in consenting to do that which I know to be wrong.

So far as the mechanical department of dentistry is concerned, there is work done in Paris equal to any in the world. What think you of two months and a half being spent upon a double set of teeth. You can hardly realize it until you examine the work, and see the delicacy and beauty of its finish, the completeness of its arrangements, the perfection of its articulation and the absolute correctness of its adaptation.

But while Paris has her true artizans in our profession, she has also her quacks. Amalgam, prepared in its worst forms, and applied in the most unskillful manner, constantly remind you, that the charlatan flourishes abroad, as well as at home.

A crowd has gathered at a window before you, you halt as you pass by; a waxen face, whose jaws move by machinery, attracts your eye, they open, and display a full set of teeth, when, ***presto change!*** the teeth have disappeared, the eyes roll, the mouth closes. Again they open, the teeth appear, and disappear, as before. You are before the door of "M. — Dentist," who will cup you, bleed you, amalgamate you, and put in ivory teeth for you, for a few francs. Some of the boys thrust his advertisments into your hands, or scatter them in your path; others, in imitation of the image before them, turn around, chatter, grin, look cross-eyed at you, and you pass on.

A few steps further on, another "gaping crowd," swell its numbers by a unit, for you are among them! insensibly, irresistibly, you stop before this quack curiosity shop. Here, on the right, are two miniature automaton figures; one sits in a chair, the other dashes at him with—is it a dirk, and is he plunging it into the heart of his victim? no, for see the gleaming key hooked to the vermilion painted tooth, and the triumphant air of *Monsieur le petit docteur,* he has extracted a tooth. Here, on the left, are various suits of moving jaws, filled with ivory teeth. Here, too, is a curious assortment of carved bone-work, mounted with ivory teeth, fantastic and fillagreed; bone bases to cover the whole palatine arch, nicely carved and perforated, so as to imitate the most delicate open worked lace, not a mere carving on the surface, but each line, or tracery, of the figure, extended quite through the base, making a complete net-work. One of these open-worked specimens has two little cupids represented on it, each clasping a bow, which meets over the centre of the palatine arch, so that if there was any nicety of adaptation, the "fancy work" could not be worn in the mouth but a few hours, without leaving a reversed impression of its figures upon the palate. And here are human teeth mounted on dentine bases, some of them filled with gold, to imitate nature.

Is that a dish of beans that little, ragged, starved urchin is gazing at so intently and wishfully, perhaps he thinks they are baked, and he is fumbling his pockets, empty as his stomach, for the *sous* that would buy them. Poor, "suffering, sad humanity," be comforted, they are not beans, but teeth, genuine, French artificial teeth, with their dead white, glistening crockery look. But here is another dish, which, for the moment, gives you a decidedly *edible* impression yourself; red beans and white, as true as ever grew in a garden, but stop, do you not see that every white is over topped with a red one; stupid that you are, they are *gum teeth!* Why do not Jones & White, Stockton & Alcock, send missionaries to this benighted land. I saw a few American teeth in Paris, but I think Dr. Evans, is the only one who uses them exclusively; certainly I saw no French teeth that would bear any comparison with those of the aboved named manufacturers.

It gave me great pleasure to meet here, among many other friends, Ehrick Parmly, D. D. S., son of Dr. Eleazar Parmly, of New York. Dr. Ehrick Parmly, has been a resident of Paris for nearly, or quite, a year, industriously pursuing a course of medical and surgical studies. Perhaps there is no city in the world, which possesses so numerous, and such superior, advantages to the student in anatomy and surgery. The medical departments of the schools are of a high order, but in the department of surgery and anatomy, Paris presents peculiar advantages to the student. There is always an abundance of subjects, easily obtained, and at the most trifling cost. In a city where bloodshed and revolutions are so common, human life is at a comparative low estimate; necessity, more often than decent humanity, finds a grave for the unfortunate poor. The hold upon life, in Paris, among the masses, is not a strong one. Suicide is very frequent, I had almost said *fashionable.* Many a bridge overstretching the Seine, is literally a "bridge of sighs." Every morning, regularly, the river yields up its dead; go any day to the dead-house, on the banks of the river, and there you see, behind a large grated door, stretched out upon as many slabs, from six to ten dripping bodies waiting to be recognized by their friends. But few of them are claimed, the cost of burial, though humble indeed, is expensive, and the medical schools confer a favor upon the city authorities by accepting of them. The hospitals, and prisons are ready, at any time, to supply you to order; be not over nice in your sensibilities, nor start if your subject sometimes come to you *warm*, you may take it for a sure consolation that it is a fresh one. Dr. Ehrick Parmly informed me, that while dissecting upon the brain, he could have a fresh one every morning if he chose; so too, with the eye, or any other organ, or part of the human body, and all too, without an additional charge upon the amount originally agreed upon for the course of lectures, itself an amount so small, I would not venture to name.

It is not so surprising then, that the French are so greatly proficient in anatomy and surgery; with such unequaled facilities, aided by their characteristic daring and energy, it would be strange indeed if they did not excel. They have nobly availed themselves of their advantage. Some of their recent works on structural anatomy, are, unqualifiedly, among the most wonderful productions of human art. In these anatomical plates, not only the general structure, but the most delicate minutiæ are represented with failfulness of delineation, a boldness of relief, perfection of execution, and freshness and life-likeness unparelleled, they startle and captivate you, while you look again and again, to find the secret by which they impress you with the idea of a vitalized and circulating organization. One artist excels another in that he *spiritualizes* his painting and makes us feel the magnetism of the soul's presence. The higher the exhaltation of this vitality of the spirit, of the subject, the more successful is the true genius in transferring it to canvas. And here we make our application. In the pursuit of earnest inquiry, the French anatomist is never limited as to the number, qualities, or condition of his subjects. They are so constantly changing, if he wills it, so constantly fresh—often warm with the vitality which has hardly yet escaped from them, I will not say *more,*

though better authority than I have said so—that his dissections are performed upon subjects whose organization is in a condition the nearest approach to the living. This *living expression,* French art has succeeded in catching and transferring upon enduring tablets, capable of indefinite multiplication.

Rash, daring, impetuous and skillful, the French surgeon dives deeper into the mysteries of the human organization, and brings up from its hidden wealth sparkling truth and gems of wonder and beauty.

If the French excel us in one department, we excel them in another. They have but little need to arrest the decomposition of a subject, nor are they skillful in preparations, herein we are greatly their superiors.

It is a matter of pride to our profession, and it has cause for congratulation that young men of such rare promise and superior qualifications, both socially and intellectually, as Dr. Ehrick Parmly, honor and represent us abroad. Dr. P. intends prosecuting his studies in Paris for about a year longer. Since my return, I see by a circular from the New York Dental College at Syracuse, that Dr. Ehrick Parmly is numbered among the faculty of that Institution for the present winter course. This is probably a mistake, as he informed me while I was in Paris, that his interests had required him to decline the appointment.

There are nearly 3000 Americans in Paris at this time ; this I believe is about the average number usually there.

The "American Medical Society," not long since established, is steadily growing in usefulness and prosperity. It numbers some seventy or eighty members, and is of a character well calculated to reflect credit upon the name it bears. The society is engaged in forming a library, and it earnestly solicits of American authors, their productions, as well as donations of medical books from others. Such gifts will prove seed sown on good ground. It is becoming in us to recollect that we have interests—growing interests, abroad, as well as at home.

I have just been informed who are the officers of the society for the present term. *President,* N. J. PITMAN, M. D., N. Carolina; 1st *Vice-President,* H. B. WALTON, M. D., Maryland ; 2d *Vice-President,* W. A. CONWAY, M. D., Louisiana ; *Corresponding Secretary,* D. R. HAYNES, M. D., Dist. Columbia ; *Recording Secretary,* R. W. GIBBS, M. D., S. Carolina ; *Treasurer,* W. E. JOHNSON, M. D., Ohio ; *Librarian,* J. WILKINS, M. D., Maryland.

W. H. DWINELLE.

